# Increased Timing Variability in Schizophrenia and Bipolar Disorder

**DOI:** 10.1371/journal.pone.0097964

**Published:** 2014-05-21

**Authors:** Amanda R. Bolbecker, Daniel R. Westfall, Josselyn M. Howell, Ryan J. Lackner, Christine A. Carroll, Brian F. O'Donnell, William P. Hetrick

**Affiliations:** 1 Department of Psychological & Brain Sciences, Indiana University, Bloomington, Indiana, United States of America; 2 Department of Psychiatry, Indiana University School of Medicine, Indianapolis, Indiana, United States of America; 3 Larue D. Carter Memorial Hospital, Indianapolis, Indiana, United States of America; National University of Singapore, Singapore

## Abstract

Theoretical and empirical evidence suggests that impaired time perception and the neural circuitry underlying internal timing mechanisms may contribute to severe psychiatric disorders, including psychotic and mood disorders. The degree to which alterations in temporal perceptions reflect deficits that exist across psychosis-related phenotypes and the extent to which mood symptoms contribute to these deficits is currently unknown. In addition, compared to schizophrenia, where timing deficits have been more extensively investigated, sub-second timing has been studied relatively infrequently in bipolar disorder. The present study compared sub-second duration estimates of schizophrenia (SZ), schizoaffective disorder (SA), non-psychotic bipolar disorder (BDNP), bipolar disorder with psychotic features (BDP), and healthy non-psychiatric controls (HC) on a well-established time perception task using sub-second durations. Participants included 66 SZ, 37 BDNP, 34 BDP, 31 SA, and 73 HC who participated in a temporal bisection task that required temporal judgements about auditory durations ranging from 300 to 600 milliseconds. Timing variability was significantly higher in SZ, BDP, and BDNP groups compared to healthy controls. The bisection point did not differ across groups. These findings suggest that both psychotic and mood symptoms may be associated with disruptions in internal timing mechanisms. Yet unexpected findings emerged. Specifically, the BDNP group had significantly increased variability compared to controls, but the SA group did not. In addition, these deficits appeared to exist independent of current symptom status. The absence of between group differences in bisection point suggests that increased variability in the SZ and bipolar disorder groups are due to alterations in perceptual timing in the sub-second range, possibly mediated by the cerebellum, rather than cognitive deficits.

## Introduction

For good reasons, an NIMH initiative is encouraging the establishment of a domain-based, dimensional classification of psychiatric illness [1], thus moving away from categorical diagnostic classification systems. It is hoped that such an approach will lead to enhanced and accelerated treatment options for psychiatric disorders since gene variants or brain circuit abnormalities are more closely linked to cognitive or behavioral abnormalities than to psychiatric diagnostic categories [2]. Interval timing deficits are implicated in several developmental and psychiatric disorders including schizophrenia, ADHD, autism. For each of these diagnostic categories there is some evidence of shared genetic risk [3], [4]. These disorders share impairments in the temporal organization of thoughts and behavior that interfere with adaptive behavior. Interestingly, cognitive processes that are deficient in these disorders are also associated with interval timing deficits including social cognition [5], [6], understanding of causality [7], and language processing [8]. In addition the linkage of interval timing to specific cognitive domains and its presence in several debilitating psychiatric disorders, interval timing is a particularly attractive because it can be used as a translational vehicle in animals to understand pharmacological mechanisms and neural circuits underlying temporal processing in humans [9]. Such knowledge is crucial for the development of novel and effective therapeutic interventions.

Few studies have examined interval timing across psychiatric diagnostic categories to date. An exception is a recent study by Penney & Meck [10] indicating that individuals at high risk for schizophrenia versus affective disorders may have modality-specific differences in clock speed relative to controls; specifically, the high risk schizophrenia group showed larger differences between auditory and visual clock speed on a temporal bisection task relative to controls, whereas the high risk affective disorder group fell between the two groups but did not statistically differ from either. The question this paper poses is whether interval timing deficits are related to symptom dimensions that cross diagnostic categories. One possible dimension is psychosis. Temporal processing abnormalities have been reported in schizophrenia over timescales ranging from milliseconds to several minutes and across a range of explicit timing tasks [11]–[18], and the associated temporal fragmentation of conscious experience and the corresponding lack of temporal organization in thoughts and behavior has been suggested to be an important contributor to the pathophysiology of schizophrenia [19]. Because of its diverse connections and uniform architecture, the cerebellum is believed to play an important role in temporal coordination of information between multiple brain regions [20], [21]. This putative temporal modulation by the cerebellum has been theorized to be disturbed in schizophrenia and could account for a multitude of symptoms of the disorder [19], [22]. With respect to psychotic symptoms specifically, temporal dys-coordination of incoming neural signals could result in translation errors that contribute to hallucinatory experiences and delusional interpretations of cerebellar output signals. For example, temporal distortions in cerebellar auditory output may cause internally generated thoughts to be interpreted as originating from an external source, resulting in an auditory hallucination [22].

Recent research has suggested that perceptual timing abnormalities may be a feature of psychosis more generally rather than of schizophrenia specifically [23]. Consistent with this idea, schizophrenia and psychotic affective disorders, including schizoaffective disorder and psychotic bipolar disorder, show more severe neurocognitive impairment than non-psychotic bipolar disorder [24], [25], which suggests that psychosis symptoms are better predictors of neurocognitive impairments than DSM diagnostic category.

To follow up on this suggestion, we examined whether perceptual timing deficits differentiate bipolar disorder with psychotic features from bipolar disorder without psychosis and to what extent mood symptoms independently contribute to timing abnormalities. Investigations of time perception in bipolar disorder for short durations, i.e., those in the second or millisecond range, are scarce. Two reports, both from the same group [26], [27], included bipolar disorder patients and compared them with a control group on time perception and in both cases comparisons have been for the supra-second range. However, mood disorders in these samples were not restricted to bipolar disorder participants only and psychotic symptoms were not explicitly examined, making results difficult to interpret. Specifically, Bschor et al. [26] reported that depressed (meeting criteria for DSM-IV major depressive episode) and manic (DSM-IV manic) patients did not differ from controls on either a 7-second time production or an 8-second time-estimation task. In a subsequent study by the same group using the same criteria [27], depressed patients over- reproduced a 6-second interval, and no differences between patients and controls were observed in a 1-second time reproduction task. To our knowledge, our recent study examining performance on a paced finger tapping task in bipolar disorder [28] was the first to examine explicit timing in bipolar disorder in the sub-second time domain; we found that bipolar disorder patients tapped faster and had increased variability that could be attributed to clock rather than motor timing variance; tapping variables were not correlated with mood symptoms, but psychosis history was not explicitly examined. Importantly, the milliseconds durations are the time range in which sensory perceptions are linked to internal cognitive and motor programs [29].

The present study set out to address the question of whether perceptual timing aberrations are associated with psychotic symptoms by studying psychosis-related phenotypes in relation to clinical populations without psychotic symptoms and to healthy controls. An additional goal was to determine whether perceptual timing deficits on this task were apparent in bipolar disorder in the absence of psychotic symptoms, given earlier evidence from our group suggesting altered clock speed and increased variability in a paced finger tapping task [28]. Specifically, the present study compared a sample of individuals with schizophrenia, schizoaffective disorder, bipolar disorder with and without psychotic features, and healthy neurotypical controls on an auditory temporal bisection task. In this task, participants classify test tones of intermediate durations to “short” or “long” anchor tones. The primary advantage of this task compared to other standard time estimation tasks is that the source of differences between groups can be attributed to either perceptual timing, i.e. “clock” variables, or to cognitive, i.e. mnemonic, factors. We hypothesized that schizophrenia, schizoaffective disorder, and bipolar disorder with psychotic features groups have significantly increased variability compared to controls and that the bipolar disorder group without psychotic features would not be significantly different from the control group.

## Methods

The study procedures were approved by the Indiana University-Purdue University Indianapolis Internal Review Board, and the study was conducted in accordance with the Declaration of Helsinki (Edinburgh amendments). Written informed consent was obtained from all participants. Obtaining informed consent involved the following steps. First, one of the investigators discussed the research study with the individual and ensured that the potential participant understood the procedures, risks, and benefits. Once an individual agreed to participate and signed the consent form, he or she was reassured once again that participation was voluntary and that it could be ended at any time without consequence. Second, prior to actually entering the study, an assessor who was not a member of the research staff once again reviewed the study and underscored the voluntary nature of participation. Inpatients who had been involuntarily committed because the severity of psychiatric symptoms had impaired their ability to manage daily affairs and impaired insight into his or her illness were not approached for the study until the symptoms had responded to treatment as judged by the patient's physician and the lack of delusions on diagnostic interview.

### Participants

There were 73 healthy controls (HC; 29M:44F), 34 bipolar disorder with psychotic features (BDP; 14M:20F), 37 bipolar disorder without psychotic features (BDNP; 16M:21F), 66 schizophrenia (SZ; 41M:25F) and 31 schizoaffective (SA; 12M:19F). Patients were recruited through physician referrals from clinics affiliated with the Indiana University School of Medicine in Indianapolis, Indiana, USA. Control participants were recruited using flyers and advertisements. Exclusion criteria for all participants included a history of neurological or cardiovascular disease, clinically documented hearing loss, head injury resulting in loss of consciousness, electroconvulsive therapy, diagnosis of alcohol or other substance dependence within 3 months, and intelligence quotient (IQ) below 70. For control participants, exclusion criteria also included a history of substance abuse or dependence, a diagnosis of any current or past DSM-IV mood or psychotic disorder, or first-degree relatives with BD or schizophrenia.

Table 1 shows clinical information for each group. Sex was unevenly distributed across groups, Χ^2^(4)  = 9.82, p = 0.04, primarily due to the difference in proportion in the ratio of males to females in the schizophrenia group compared to the other 3 clinical groups (BDNP, BDP, SA). Age did not differ across groups (F(4, 234) = 0.47, p = 0.76).

**Table 1:** Clinical and IQ information for each group.

	BDNP	BDP	SZ	SA	Controls
PANSS total score			56.5 (12.6)	54.4 (11.8)	—
* Positive*			15.3 (5.6)	14.9 (4.5)	—
* Negative*			14.2 (5.0)	12.2 (3.8)	—
* General*			26.9 (6.5)	27.3 (6.4)	—
YMRS total score	8.5 (10.0)	15.9 (14.1)			
MADRS total score	9.6 (9.7)	9.6 (11.6)			
WASI IQ	108 (16)	102 (14)	92 (14)	95 (14)	110 (14)

Diagnostic status for the schizophrenia group was determined using the Structured Clinical Interview for Diagnostic and Statistical Manual of Mental Disorders-IV Axis I Disorders, Patient Version (SCID-I/P) [30] sections for mood disorders, psychotic disorders, and substance abuse disorders, as well as chart review. Kappa inter-rater reliability in our lab has been 0.95 for schizophrenia versus mood disordered, or other diagnoses in patients who have been prescreened for showing psychosis. Control participants were interviewed using the non-patient version of SCID-I/NP [31] sections for mood, psychotic, and substance abuse and the SCID II [32] to exclude psychiatric disorders. All diagnostic and clinical interviews occurred within 14 days of the temporal bisection task. Mood symptoms in participants with bipolar disorder were assessed using the Young Mania Rating Scale (YMRS; available for 28 BDP and 31 BDNP) [33] and the Montgomery-Asberg Depression Rating Scale (MADRS; available for 27 BDP and 31 BDNP) [34]. Schizophrenia and schizoaffective participants' symptoms were assessed using the Positive and Negative Syndrome Scores (PANSS; available for 51 SZ and 22 SA) [35]. Finally, WASI IQ was available for 67 HC, 32 BDP, 37 BDNP, 61 SZ, and 30 SA. Clinical and IQ information can be found in Table 1. The number and percentage of each group taking the major classes of psychotropic medication are listed in Table 2. Complete medication information was not available for 2 BDP, 2 SZ, and 1 SA.

**Table 2:** Numbers and percentages of major psychotropic medications prescribed across groups.

Psychotropic Medications				
	BDNP (N = 37)	BDP (N = 32)	SZ (N = 64)	SA (N = 30)
***No psychotropic medication***	14% (N = 5)	22% (N = 7)	9% (N = 6)	13% (N = 4)
***Atypical antipsychotic***	68% (N = 25)	72% (N = 23)	77% (N = 49)	80% (N = 24)
***Typical antipsychotic***	3% (N = 1)	9% (N = 3)	23% (N = 15)	7% (N = 2)
***Anticonvulsant***	27% (N = 10)	38% (N = 12)	11% (N = 7)	23% (N = 7)
***Antidepressant***	16% (N = 6)	28% (N = 9)	23% (N = 15)	40%(N = 12)
***Anticholinergic***	0% (N = 0)	0% (N = 0)	11% (N = 7)	5% (N = 5)

*No control participants were taking psychotropic medication. Medication information was not available for 2 BDP, 2 SZ, and 1 SA.

### Task procedure

Tone stimuli (880 hz) consisted of anchor tones with durations of 300 and 600 ms, along with five arithmetically spaced intermediate durations of 350, 400, 450, 500, and 550 ms.

During the test phase of the experiment, tones were classified according to their perceived similarity to the short (i.e., 300 ms) or long (i.e., 600 ms) anchor values. To address potential difficulties related to task comprehension, a concrete procedural context related to bird classification was adapted from Elvevåg and colleagues [15].

The task procedure was divided into training, practice, and test phases. The experiment began with a training phase in which the short and long anchors were paired with a small (1.84×1.92 in.) and a large (3.60×3.78 in.) bird silhouette, respectively. To ensure that participants had learned the anchor durations, six presentations of each anchor were randomly administered within a 12-trial practice block in the absence of the associated bird silhouette. Following each presentation, participants received on-screen instructions to press the “Short” key if the sound was made by the small bird and to press the “Long” key if the sound belonged to the big bird. Visual feedback (i.e., “correct” or “incorrect”) was provided after each response, and correct responses were associated with a monetary bonus of 10 cents. A 1 s inter-trial interval separated feedback offset and stimulus presentation. The practice phase was repeated in 12-trial blocks until an accuracy level of 75% or greater was reached: the session was aborted if 75% accuracy had not been achieved after three practice blocks.

The test phase of the experiment was presented in three blocks of 35 trials each (five presentations of each auditory duration). Participants were asked to classify each auditory stimulus as either “Short” or “Long” based on their perceived similarity to the sounds made by the small or large bird. Because the bisection task is an assessment of subjective time perception, response accuracy could only be determined for the anchor durations during the test phase. Each correct classification of the short and long anchors earned participants a reward of 10 cents, for a possible bonus of $3.00. To help ensure that the participants understood the task, a practice block consisting of one presentation of each stimulus was administered immediately prior to the test phase to allow participants to ask questions and become familiar with the procedure.

Each test block was preceded by a short (<5 min) rest period. To minimize memory demands for the anchor values, the short and long anchors were presented and paired with the small and big bird silhouettes, respectively, prior to the commencement of each of the three test blocks.

### Behavioral data and analysis

The proportion of long responses, p(long), made to the anchors and intermediate signals were quantified separately for each participant and duration condition. The proportional data can be plotted as a function of signal duration to yield a psychometric response curve that is typically sigmoidal in form, indicating a near absence of long responses to signals that fall close to the short anchor value, to a predominance of long responses as signals come to approach the duration of the long anchor. Sigmoidal functions were fit to the proportional response data from each participant using the regression feature of SigmaPlot 9.0 (Systat Software, Inc., San Jose, CA), which employs a least squares method to estimate equation parameters and identify the durations that correspond to p(long) values of 0.25, 0.50, and 0.75 from the fitted sigmoidal curve.

The duration at which the proportion of long responses was equivalent to 0.50 for each duration condition was identified as the bisection point, or the duration at which short and long responses occurred with equal probability. In addition to the bisection point, the values derived from the fitted sigmoidal functions were used to calculate the difference limen (DL) and Weber fraction (WF), which represent the slope of the psychometric response curves and can be interpreted as an index of timing variability. The DL is calculated as one-half the difference between the durations corresponding to p(long)  = 0.75 and p(long)  = 0.25 ((0.75–0.25)/2), where smaller values indicate steeper slopes and greater temporal precision. The WF is computed by dividing the DL by the bisection point, which normalizes the DL values with respect to the timed durations. Thus, the WF provides an index of Weber's Law (i.e., a constant coefficient of variation of subjective time across various temporal durations) by allowing for a direct comparison of timing variability across various anchor pairs.

All statistical analyses were conducted using SPSS 21.0. Non-parametric Kruskal-Wallis tests with Group (SZ, SA, BDP, BDNP, HC) as a between-subjects factor were used to analyze differences between groups when assumptions of traditional analysis of variance (ANOVA) were violated, i.e. the Levene's test for homogeneity of variance and the Shapiro-Wilks test for normality were significant (p<0.05), as was true for all dependent variables for the temporal bisection task; moreover, nonparametric tests are more resilient to unequal sample sizes such as those in the present study. Results were considered significant if they were below p<0.05. Planned Mann-Whitney post-hoc tests comparing clinical groups with the control group were conducted using a Bonferroni corrected alpha level of 0.013 (0.05/4 = 0.0125 = 0.013).

The effects of clinical symptoms were evaluated using planned bivariate Pearson correlations of primary dependent variables (bisection point, DL and WF) with YMRS and MADRS scores for the bipolar disorder groups and with the PANSS for the schizophrenia spectrum groups (schizophrenia and schizoaffective disorder).

## Results

### Practice accuracy

All participants were able to differentiate between the “short” and “long” anchor tones with a 75% accuracy rate by the end of the practice session. Practice accuracy did not differ across groups, H(4) = 4.27, p = 0.371. However, there were differences between groups with respect to how many participants took 2 practice blocks to achieve the 75% criterion, X^2^(4) = 12.28, p = 0.02; this effect was driven by the SZ group, the only group whose observed count exceeded the expected count (expected:observed = 5:11).

### Bisection point

**Figure 1:**
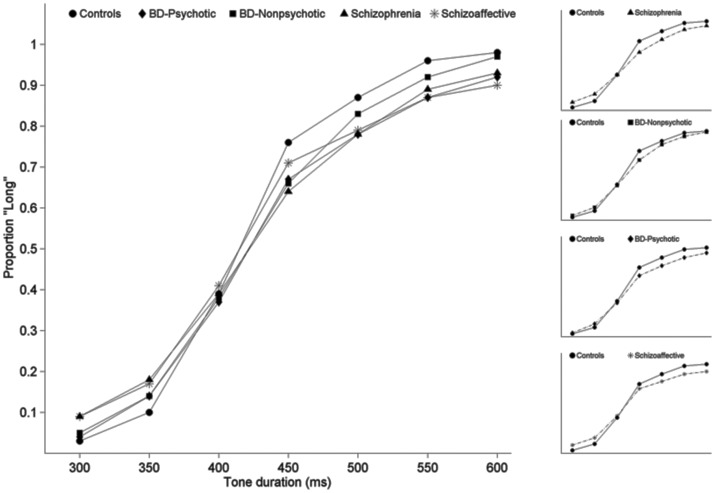
Psychometric response curves for each group composed of the proportion of long responses as a function of signal duration. Smaller insets to the right show each clinical group individually as compared to the control group.

**Figure 2:**
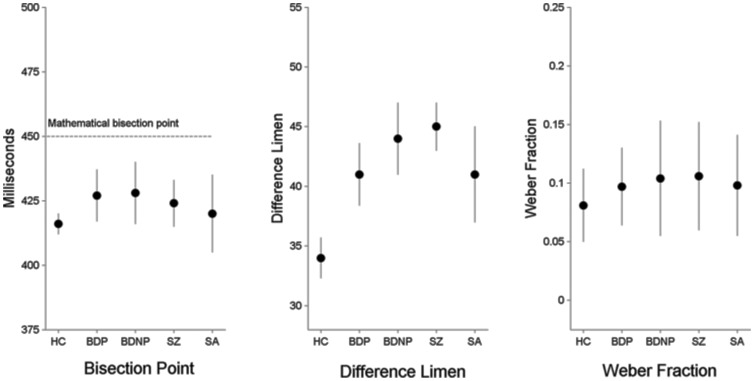
Means and standard deviations for each group for the bisection point (left panel), difference limen, (center panel) and Weber Fraction (right panel). No differences existed between groups on bisection point, although for all groups the perceived bisection point occurred earlier than the mathematical bisection point. SZ, BDP, and BDNP had increased temporal variability on both the difference limen and the Weber Fraction compared to controls. The SA group was not significantly different from controls.

The proportion of Long responses for each of the test durations and the resulting psychophysical response functions for each of the 5 groups are plotted in Figure 1, where they are largely overlapping. Likewise, the mean bisection point for each group, plotted in the left panel of Figure 2, is similar across groups. Consistent with these observations, the groups did not differ statistically on bisection point (H(4) = 1.61, p = 0.81).

### Response variability

Examination of the 4 panels on the right side of Figure 1 shows that in general the psychophysical functions the clinical groups had flatter slopes compared to controls. This impression was confirmed by a significant effect of Group on DL (H(4) = 17.53, p = 0.002). Post-hoc tests using Mann-Whitney tests showed increased variability in the SZ (p<0.001), BDP (p = 0.009) and BDNP (p = 0.003) groups compared to controls; however, the SA group did not differ statistically from HC (p = 0.136). There was also a significant effect of Group on WF (H(4) = 15.64, p = 0.004), which compares variability for each group across different durations. Follow-up Mann-Whitney tests showed the same pattern of significantly increased variability in the SZ (p<0.001), BDP (p = 0.013), and BDNP (p = 0.007) groups compared to HC; the SA group was not statistically different from controls (p = 0.058).

### Clinical symptom measures, IQ, and planned correlations

At the time of testing, mean YMRS and MADRS ratings for the bipolar disorder groups were not significantly different (p>0.05). Within the entire bipolar group, the YMRS and MADRS were not significantly correlated with the bisection point, DL, or DF.

Likewise, PANSS scores for the schizophrenia spectrum groups (SZ and SA) were not significantly different on total score, nor were the positive, negative, and general symptom dimensions significantly different (all p>0.05). PANSS positive, negative, general, and total scores were not significantly correlated with any of the primary dependent variables (all p>0.05).

WASI IQ showed main effect of Group, F(4) = 16.48, with post-hoc test indicating that significant differences existed between controls and BDP, SZ, and SA (all p<0.013) but not BDNP (p = 0.07). All WASI IQ group means were well within the normal range, however (see Table 1). Within-group correlations of IQ with bisection point, DL, and WF were not significant (all <0.05).

## Discussion

The hypothesis of this study was that schizophrenia, schizoaffective disorder, and bipolar disorder with psychotic features (but not bipolar disorder without psychosis), would show increased temporal variability compared to healthy controls. Results partially supported this hypothesis. The results indicated increased temporal variability on both the DL (difference limen) and WF (Weber Fraction) in schizophrenia and in bipolar disorder. Two unexpected results emerged. First, increased variability was apparent in the BD group irrespective of whether a history of psychotic features was present. Second, the schizoaffective group was not statistically different from controls on either variability measure. These results indicate that factors other than the simple presence or absence of psychotic features contribute to timing variability. Furthermore, the fact that the schizophrenia group showed the largest increase in variability but the schizoaffective group did not have significantly increased variability compared to controls suggests that temporal perception is relatively more preserved in this phenotype.

In recent years evidence has accumulated that psychosis is a defining feature that contributes to cognitive impairment, crosses diagnostic boundaries to encompass schizophrenia spectrum and psychotic affective disorders, and differentiates psychotic from nonpsychotic bipolar disorder [24], [36]. It has also been reported that individuals with schizoaffective disorder have more cognitive impairment and poorer functional outcomes compared to bipolar disorder without psychotic features [37]. The current results found significantly increased variability in timing in bipolar disorder whether psychosis was present or not, which, along with the absence of significantly increased variability in schizoaffective disorder, suggests a more nuanced view of the relative contributions of psychotic versus affective symptoms to brain mechanisms underlying temporal processing, which has been argued to be an important substrate of neurocognitive functioning [19].

The lack of between group differences in bisection point is important because it strongly suggests that perceptual factors were likely the primary factor contributing to the observed differences in timing variability. This inference derives from the fact that, because temporal bisection requires the comparison of two tones, alterations in clock speed would cause the relative perceptions of the duration of the two comparison tones to be rescaled according to each individual's internal clock. Hence, clock speed differences alone should not affect the location of the bisection point. If the bisection point were different across groups it would suggest that cognitive factors could have influenced results. For example, if memory of anchor durations were impaired, the temporal relationships of anchor-probe comparisons would be distorted and bisection point would be shifted. This similarity of bisection points across groups suggests that perceptual alterations were the primary factor contributing to increased variability in bipolar disorder and schizophrenia.

With respect to mechanisms underlying the observed increases in variability, it is generally accepted that the frontal cortex, basal ganglia, and cerebellum are integrally involved in time perception, with a consensus emerging that different timescales utilize different neural circuits [38], [39]. A recent meta-analysis of 41 functional neuroimaging studies of perceptual and motor timing used a robust activation likelihood estimation algorithm and found strong support for the theory that sub-second and supra-second durations depend on somewhat distinct neural networks, with the former more likely to recruit subcortical structures such as the basal ganglia and cerebellum and the latter more likely to activate cortical structures such as the supplementary motor area and prefrontal cortex; moreover, activation of the cerebellum was consistent across motor and perceptual timing tasks [40]. The conclusions from that meta-analysis support the proposal that the cerebellum serves as a timekeeper for brief durations [41]. The finding that the cerebellum is activated predominantly during sub-second tasks is consistent with other suggestions that this structure may be critical for the encoding of sub-second time intervals [38], [39].

The foregoing evidence fits well with the influential cognitive dysmetria theory of schizophrenia put forward by Nancy Andreasen [19] which highlights the potentially important role of the cerebellum by suggesting that disturbances in temporal processing stemming from dysfunction of the cerebellar node of the cortico-cerebellar-thalamic-cortical circuit may provide a unitary model of schizophrenia that could produce the diverse symptoms of schizophrenia ranging from hallucinations to cognitive impairment. This model has been suggested with schizophrenia specifically in mind, and a growing literature supports cerebellar abnormalities in this disorder. For example, postmortem and imaging studies report reduced volume of the CB in chronic [42]–[45], neuroleptic-naïve [46], adolescent [47], first-episode [46], [48], [49], and childhood-onset [50] SZ, as well as reduced bilateral hemispheric volume in first-episode SZ [51]. Postmortem studies also have found reduced size and density of Purkinje cells in SZ [52]–[54]. In addition, functional neuroimaging studies have reported abnormal CB blood flow at rest [55]–[57], and during cognitive tasks [58]–[60] in SZ patients.

Importantly, it should also be noted that the cerebellum is also increasingly implicated in bipolar disorder, including findings of neurotransmitter alterations [61], [62], and reduced white [63] and gray [64] matter. Moreover, lesions to the cerebellum can cause disturbances in mood including mania, depression, and mood lability [65]. Finally, our group has documented abnormalities in bipolar disorder on several tasks for which the cerebellum is critical including classical delay eyeblink conditioning [66], paced finger tapping [28], and postural sway [67]. This evidence supports a possible role for the cerebellum in bipolar disorder and suggests that dysfunctional cerebellar circuitry may contribute to timing deficits observed in the present study.

Strengths of the current study include relatively large sample sizes and the inclusion of 4 clinical diagnostic categories with differing degrees of psychotic versus affective symptoms. We found no relationships of timing variability to mood symptoms in bipolar disorder or PANSS ratings in schizophrenia spectrum disorders. However, with respect to clinical symptoms at the time of testing, a weakness of our study is the lack of concurrent psychosis and mood state information. Specifically, bipolar disorder patients had YMRS and MADRS information available that provided an index of their current mania and depression symptoms, respectively; schizophrenia and schizoaffective individuals had PANSS data available which assessed their current psychosis-related symptoms. However, a mixture of psychotic and mood symptoms of differing magnitudes affect these disorders, so we were unable to completely assess the contributions of these symptom domains within each group.

Overall, the reported results suggest that psychotic and mood symptoms contribute to increased timing variability and that performance differences on timing tasks for these disorders can be attributed to perceptual, or “clock” alterations rather than differences in memory performance, as indicated by the lack of between groups differences on bisection point. Timing abnormalities in the range reported here are consistent with abnormalities in circuits in which the cerebellum participates, although multiple brain regions are likely to be involved. In future work, complete information about the presence or absence of manic, depressive, and psychotic symptoms across these diagnostic groups could provide important information about the relative contributions of each of these symptom domains to timing variability. However, the lack of correlations between timing variability and the ratings that were available suggest that more enduring traits and patterns of symptoms over time, rather than transient symptoms, are more likely to be associated with temporal processing abnormalities.
